# Visioning future treescapes in upland landscapes: using deliberative processes to understand values and land-use preferences of local stakeholders

**DOI:** 10.1080/26395916.2025.2497823

**Published:** 2025-05-09

**Authors:** Alix Syder, Susan Baker, Euan Bowditch, Sheena Carlisle, Tom Finch, Melissa Minter, Natasha Constant

**Affiliations:** a Centre for Conservation Science, The Royal Society for the Protection of Birds, Centre for Conservation Science, Sandy, England; b School of Social Sciences, Cardiff University, Cardiff, Wales; c Institute for Biodiversity and Freshwater Conservation, University of Highland and Islands, Inverness, Scotland

**Keywords:** Zuzana V. Harmackova, Ecosystem services, participatory scenario planning, land-use, net zero, UK

## Abstract

UK environmental policy places an increasing emphasis on large-scale land-use change, with tree-planting objectives set to contribute towards meeting legislated climate and environmental targets. Upland landscapes might expect to see disproportionate change because of the perception that opportunity costs (e.g. from foregone agricultural activities) are low. However, without considering the preferences of local stakeholders, delivery may be misaligned, underlying conflicts not considered and local actors alienated. Land-use preferences are shaped by the values stakeholders attribute to landscapes, and broader contextual factors, both biophysical (i.e. climate change) or institutional (i.e. land-use policy and financial instruments). This paper explores the relationship between values, contextual factors, and land-use preferences, by applying Participatory Scenario Planning (PSP) to design future land-use visions of local stakeholders in two upland landscapes in England (North Pennines and Dales) and Wales (Elenydd). The paper address two overarching research questions (1) How do different stakeholders value upland landscapes? and (2) How does context shape stakeholders’ decisions regarding future land-use? Whilst our results show a greater potential for treescape expansion in the uplands than expected, underlying nuances of land-use preferences demonstrate challenges to treescape expansion here. Our approach also highlights the importance of taking into account contextual factors when examining land-use preferences, for example climate change as a positive driver for on-farm treescape measures, whereas regulatory context limit stakeholders’ ambition for change. Only by understanding these complexities through deliberative processes can future treescape expansion at local scales achieve the best outcomes for people and nature.

## Key policy highlights

- Values shape stakeholder land-use preferences, considering this in decision-making may reduce conflicts arising from land-use change.
- Land-use preferences vary as a result of different landscapes’ contexts.
- Large-scale tree planting and current delivery mechanisms tended to be less compatible with local values and preferences.
- A focus on natural processes for treescape expansion tends to be seen as more in keeping with the landscape’s character in upland areas.

## Introduction

The UK Government has pledged to reach net zero by 2050 with transformative objectives for decarbonising society and enhancing the natural environment (Climate Change Committee, 2020). Nature-based solutions could contribute to achieving national net zero targets, although they cannot replace wider cross-sectoral emissions reductions (Bradfer‐Lawrence et al. 2021; Finch et al. 2023). Expanding future treescapes is considered a key measure for delivering UK net zero objectives (Climate Change Committee, 2020), including creating 30,000 hectares of woodland per year by 2024 (UK Government 2021). ‘Future treescapes’ broadly encompasses ‘landscapes with trees’, capturing a range of forms and scales in which trees can be integrated into the landscape, such as agroforestry, wood pasture, hedgerows and woodland (Rotherham 2013; Kirby 2018).

The UK’s upland landscapes have a typically low agricultural output, which has led to assumptions that future land-use change might occur disproportionately in these ‘marginal’ areas (e.g. National Food Strategy 2021). However, the uplands do not present a blank canvas for land-use change, and treescape expansion is likely to be contested due to existing farming, sporting, conservation and recreational uses. Sustainable and equitable treescape expansion therefore requires an understanding of the land-use preferences of local stakeholders within these landscapes. Local stakeholders experience and use landscapes in different ways, attributing different values to landscape features and associated ecosystem services (ES). While acknowledging that the investigation of values is complex and the term itself has a variety of definitions across disciplines, this paper understands values as ‘opinions and judgements about the importance and meaning of something’ (Himes et al. 2024). Recent research, acknowledging the socio-cultural dimension of land-use decision-making, has shown that values guide land-use practices, including preferences for land-use changes in response to threat or crisis (Hodel et al. 2024). These values are deeply rooted in a community’s culture, and form the customs, guide the behaviours, and shapes the attitudes of its members (Mifsud et al. 2023). Values are key to local identity and history, shaping sense of place and the perceptions of the aesthetic worth of landscapes. Proposed landscape change can result in fears over the loss of cultural values and estrangement from what is to be newly created. This cultural dimension is often overlooked in conservation initiatives (Leduc and Von Essen 2019), and requires us to challenge perspectives of nature restoration through multiple alternative lenses (Deary and Warren 2017). This highlights the need to establish policy frameworks that account for the diversity of nature’s values to people across different cultural context, leading to an advanced understanding of how social values, embedded in institutional context, shape social preferences (Hodel et al. 2024).

**Figure 1.**
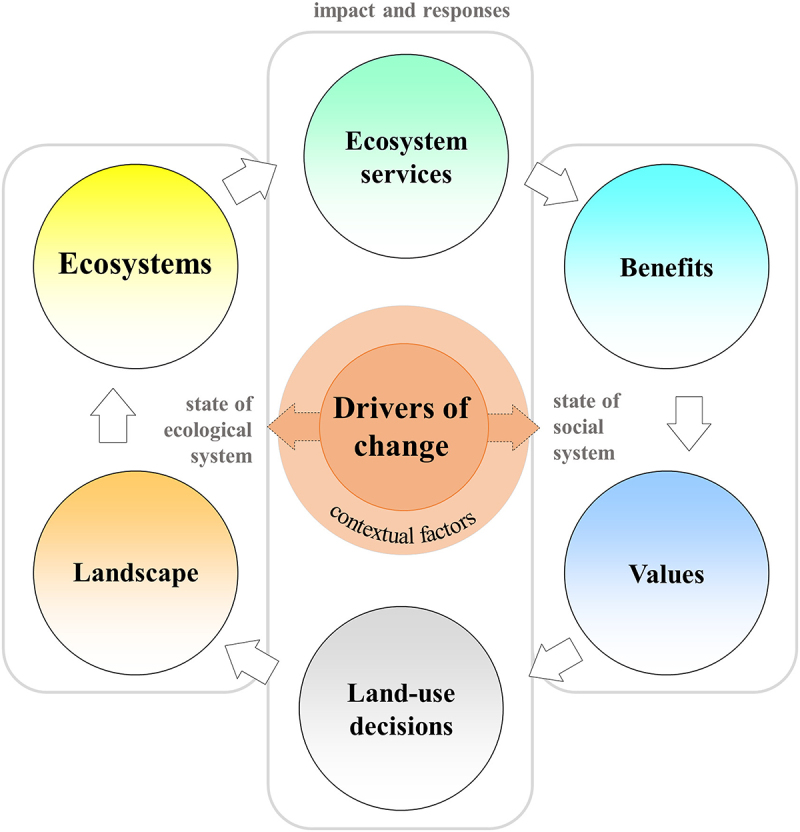
Modified ecosystem service cascade framework presented by Fedele et al. (2018). ‘Reducing risks by transforming landscapes: cross-scale effects of land-use changes on ecosystem services’. PLOS ONE 13(4).

Fedele et al. (2018) developed an adapted version of the ES cascade; that is the relationship between ecosystems, services, benefits and values (Figure 1). The functional characteristics of ecosystems give rise to services (ES) (provisioning, regulatory, cultural or supporting) and benefits (e.g. contribution to human wellbeing), from which value is attributed (Haines-Young et al. 2007). Values influence our decisions which reinforce feedback loops between the social and ecological systems, however Fedele et al. (2018) then considers how contextual factors, those external drivers both natural or anthropogenic, may also influence our land-use decisions and therefore preferences for one future state over another (Figure 1). Fedele et al. (2018) argues that stakeholders themselves will adjust their land-use choices according to their individual perception of these contextual factors; with examples of people in Indonesia changing land uses to adapt to multiple environmental risks (Fedele et al. 2018) or farmers altering land management choices in response to climate change (Dorning et al. 2017; Eitzinger et al. 2018). Considering different value expressions can help understand why perspectives on nature and nature ´s contributions to people are divergent (sources of conflict, disagreement) or convergent (sources of collaboration, legitimation, alliances) (Anderson et al. 2022). In particular, the role of culture in land-use decision making is underexplored (Hodel et al. 2024), we therefore seek to address how relationships between values and land-use relate, under local context, to our preferences in future land-use decisions.

To date, understandings of stakeholder values for treescape expansion have not simultaneously explored the effects of both values and contextual factors on shaping land-use preferences. Instead studies have focused on attitudes toward woodland expansion (Nijnik et al. 2010, 2017; Urquhart et al. 2012; Duesberg et al. 2013; Lawrence and Dandy 2014; Iversen et al. 2022; Bowditch et al. 2023), contestations surrounding land-use planning, direct management decision-making (Van der Wal et al. 2014; Eastwood et al. 2024) and compatibility with other land-use practices (i.e. agriculture, recreational, forestry and sporting) (Burton et al. 2019; Fitzgerald et al. 2021).

Future scenarios and pathways for sustainability are largely driven by people’s decisions and actions that are underpinned by a diversity of motivations and values (Sandström et al. 2020). Participatory Scenario Planning (PSP) is a collaborative approach where researchers and stakeholders develop scenarios to explore possible futures, and interrogate their associated challenges, while incorporating local knowledge and experiences into scenario design (Reed et al. 2013; Malinga et al. 2013; Oteros-Rozas et al. 2015; Metzger et al. 2017). Its use has been bolstered by global science-policy initiatives like the Millennium Ecosystem Assessment (Millenium Ecosystem Assessment 2005) and the Intergovernmental Platform for Biodiversity and Ecosystem Services (IPBES), which adopted participatory scenarios to help decision-makers evaluate the potential impacts of various policy options (IPBES 2016). The incorporation of values-led assessments in PSP have been few; a knowledge gap we aim to address and expand upon. To date, Rawluk et al. (2018) adopted ‘value-based scenario planning’ to understand key value tensions in social-ecological planning and management settings. Similarly, Harmáčková et al. (2022) applied the Life Framework of Values and the Three Horizons Framework to explore the linkages between individual values and development pathways for future action. In contrast, we explore values assessments in more deliberative decision-making contexts where defined stakeholder groups work collaboratively to co-create future scenarios through a consensus-building approach.

In this paper, we apply one of the first empirical studies to assess linkages between stakeholder values, context specific factors and future land-use preferences through a deliberative PSP approach to treescape expansion in upland landscapes. We address two overarching research questions (1) How do different stakeholders value upland landscapes? and (2) How does context shape stakeholders’ decisions regarding future land-use? This paper first sets out our methodological processes detailing the deliberative PSP approach to constructing future land-use visions within two upland landscapes. A thematic analysis is then conducted of values and contextual factors discussed by stakeholders during the articulation of their land-use preferences. The presentation of the research findings compares the influence of values and contextual factors between stakeholder groups and discusses the resulting synergies and differences between their future land-use preferences. From this we draw conclusions regarding the resulting opportunities or challenges for upland treescape expansion.

## Methods

### Case study sites

Our study landscapes currently host relatively low levels of tree cover (2.5% in NPD and 9.7% in the Elenydd) and relatively high coverage of existing ecologically designated sites, common land and complex land-use histories, thus exemplifying the challenges and opportunities for upland treescape expansion. Upland landscapes might expect to see disproportionate change because of the perception that opportunity costs (e.g. from foregone agricultural activities) are low, however they are also highly culturally embedded having been shaped by traditional practice. This has resulted in social conflicts relating to land-use change which threaten their cultural fabric, especially felt in the UK amongst low productive uplands where culturally embedded sheep farming predominates (Wynne-Jones et al. 2018). Our landscapes are not necessarily representative of the wider uplands of England and Wales, which can vary substantially in land-use, vegetation, geology, and history.

#### Elenydd, Wales

**Figure 2.**
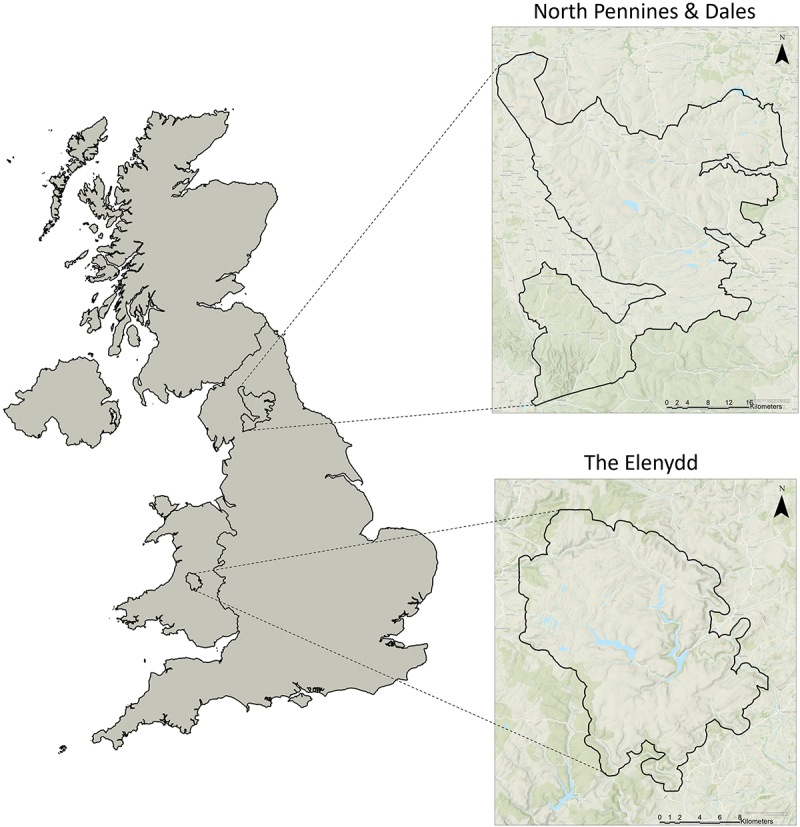
Boundaries of focal landscapes North Pennines and Dales (England) and The Elenydd (Wales). Basemap: GB Topographic Hillshade (Esri UK, 2024).

The Elenydd (Figure 2) includes the Dŵr Cymru Welsh Water estate (managed by the Elan Valley Trust) and part of the adjoining National Trust estate. The Elan Valley was shaped by the compulsory purchase of land in 1892 under the Birmingham Cooperation Act for the creation of reservoirs. The Elenydd includes important Special Area of Conservation (SAC) woodland managed by Celtic Rainforests on behalf of Natural Resources Wales (NRW).

#### North Pennines and Dales (NPD), England

The NPD case study (Figure 2) follows the Heart of the Pennines Forest project area. Launched in January 2023 by the North Pennines National Landscape Authority, aiming to increase tree cover. The landscape encompasses the lower half of North Pennines National Landscape and the north-west section of the Yorkshire Dales National Park. The NPD has several large grouse shooting estates, private forestry, a Ministry of Defence training estate as well as a one of England’s largest National Nature Reserve and other designated sites.

### Stakeholder selection

We conducted a stakeholder mapping exercise to identify participants who could represent a wider interest group through their association with an organisation or group (i.e. a recreational group, farmers association, public body or trust). Activities included a search of existing projects within the landscapes, snowballing of relevant organisations, and informant interviews with local conservation staff. Identified stakeholders were screened via the following criteria:

1. Currently living or working within the landscape – stakeholders were excluded if their focus expanded across a broader geographic remit.
2. Involvement (associated activities or job role) at a ground level – stakeholders were excluded if they operated at a higher strategic level within organisations.

Shortlisted stakeholders were categorised into four major interest groups (Table 1), categories were defined based on our knowledge of land-use within upland areas. A pre-survey of attendees, capturing details of their relationship to the landscape, job role and broad interests, helped confirm participants’ suitability to their assigned grouping. Attendees totalled 19 in NPD and 12 in Elenydd with 3–6 stakeholders per group. In the Elenydd, a separate meeting was held with farming stakeholders who could not attend the day-long workshop.

**Table 1.** Definitions of local stakeholder interest groups.

Conservation	Main interests are in nature recovery and conservation. May or may not own or manage land such as nature reserves. Typically represented by NGOs, not for profits or public bodies.
Land	Involved in the management or ownership of large estates, or industry-based professions (water, forestry, grouse etc). An economic or industry-based interest in land-use, private or public estates.
Access	Members of the community who access the land, but do not own or manage land. May include non-landowning residents, community groups and recreational users.
Farming	Main interest is in farming, may directly own land or manage as tenant farmers.

### Data collection

A two-part workshop series was conducted as part of a larger PSP exercise. This paper reports on the visions developed by local stakeholders during the first round of workshops.

Participants received an information sheet which explained research objectives, and all signed their informed consent to participate in the study, approved by the RSPB Ethics Committee on 11 May 2023 (reference: HEC_39_STAND). The workshops followed a series of activities to elicit stakeholders’ land-use values and prompting questions to create a 2050 vision for the landscape (Table 2). PSP offers several advantages for assessing values and group-level dynamics in deliberative settings, by creating a reflective space to encourage dialogue among diverse stakeholders on management and land-use decisions (Oteros-Rozas et al. 2015), and uncertain or sensitive topics, to foster deeper recognition of value conflicts (Kenter et al. 2016; Rawluk et al. 2018; Kiatkoski Kim et al. 2022). Deliberative assessments of ESs enable the acknowledgement of the reciprocal relationship between people and nature, accounting for complex non-material values (Constant and Taylor 2020) and the plurality of benefits derived from diverse worldviews and context-specific perspectives (Fish et al. 2016; Lyver et al. 2016).

**Table 2.** 2050 vision attributes and descriptions.

Vision attributes	Description
Vision statement	An overarching vision statement capturing their desired vision for the future.
Ecosystem services	Selection of top 5 ecosystem services (the benefits people obtain from the landscape). The term nature’s benefits to people was also used, depending on which term stakeholders were most comfortable with.
Land-use change	A specific change in land cover, use or management (e.g. woodland creation, peatland restoration, meadow restoration). See Supplementary Material, Table 3 for full list of land-use change types and their descriptions.
Spatial criteria	A ’rule’ defining where a specific land use should (or shouldn’t) be located within the case study landscape (e.g. no tree planting on peat soils; or prioritise woodland creation next to existing woodland)
Wider change	Additional changes outside the list of land-use changes provided e.g. housing and infrastructure, solar or wind energy, permissive footpaths.

**Table 3.** Definitions of value themes influencing land-use preferences.

Values	Descriptions
**Economic**	Monetary value based on market prices or indirect economic valuation methods. May include employment, public payments through land management schemes, or economic value of resources.
**Ecological**	Integrity of the ecological condition and function of ecosystems for the benefit of nature.
**Social**	Non-material values relating to society, community and people benefits.
People and community	The importance of people within the landscape, the sense of community and the co-existence of people and nature.
Services	The regulatory services which the land can offer for the benefit of people, such as carbon sequestration, water quality and flood management.
Access and recreation	Opportunities for physical exercise recreation and outdoor enjoyment.
Food and farming	Food production and the wider farming system.
Sustainability	Sustainable actions, industries or resource use; having no net negative impact on the landscape or people and ensuring its long-term viability.
**Cultural**	Non-material values that people obtain from an ecosystem through the culture and heritage of the landscape.
Stewards	Farmers’ role as custodians of the landscape across generations.
History and Heritage	The historical features of a landscape, and the heritage it holds.
Aesthetic	Visual characteristics of a landscape.

All activities were conducted within stakeholder interest groups (Table 1) and were audio recorded with a facilitator and note-taker present. Participants had access to maps of current tree cover, land cover, designations, geographic features, satellite imagery and Ordnance Survey mapping, in addition to cue cards describing 11 land-use options (see Supplementary Material; Table S3). A full list of stakeholders’ 2050 visions is presented in Supplementary Material; Table S4.

### Data analysis

All audio recordings were transcribed verbatim, speakers anonymised, and transcriptions coded using NVivo software. A thematic content analysis was conducted to identify (1) *value themes* (Supplementary Material, Table S1) and (2) *contextual factors* mentioned by stakeholders during deliberation (Supplementary Material, Table S2), as per Fedele’s framework (Fedele et al. 2018). The thematic analysis consisted of an initial intuitive code, as is recommended when exploring value-based data to organically generate new themes (Constant and Taylor 2020; Teff-Seker et al. 2022), before a subsequent focused code to eliminate, re-define or merge codes. During refinement we adopted a hybrid approach where codes were iteratively reviewed against existing values and ES frameworks (Haines-Young et al. 2007; Pascual et al. 2017; Breyne et al. 2021). Transcripts for each landscape where initially coded separately however, during refinement sufficient overlap was found to warrant aligning themes between the two landscapes. Variation in sub-themes between landscapes and groups have been retained in the full breakdown (Supplementary Material Table S1-S2). Coding produced 10 value themes and 7 contextual factors (Supplementary Material, Tables S1 and S2).

## Results

### Value associated with stakeholders’ future visions

Four overarching value themes (ecological, economic, social and cultural) were represented in stakeholders’ future visions, with social and cultural values sub-divided to present their wider complexity (Table 3).

#### Economic

Land and Farming stakeholders in NPD and Access stakeholders in the Elenydd referenced the economic value of food and animal products as an income source for local livelihoods in their final vision (Supplementary Table S4). All stakeholders emphasised the economic value of future employment opportunities to retain people in the landscape but, delivered through different land-use mechanisms; such as tourism, farming or ecological restoration work.

Whilst farmers in both landscapes discussed economic values surrounding compensation for land management changes, deeper socio-cultural values were driving their land-use preferences. Farmers highlighted that they may not react quickly to economic motivations alone, often taking more caution in their decision making given the longer timescales in which their decisions operate on. Access stakeholders discussed the ‘idealism’ of traditional subsistence farming, but believed farmers were motivated by economic concerns which did not align with how they, as Access stakeholders, valued the landscape ‘*So ultimately the money is driving it. If the money was there to help do it, then there would be more cases [of nature-friendly farming]’ (Access, Elenydd)*.

Land stakeholders in the Elenydd saw commercial forestry as a financing mechanism for re-investing into nature restoration projects. Tree planting could offer future financial security for landowners ‘*to give sustainability to his farm in the future as the grants go’ (Land, Elenydd)*. Overall, however, future treescapes were not valued for their economic benefits, and commercial forestry was only incorporated by some Land stakeholders in NPD. Economic value instead was attributed to other land uses through farming, tourism, industry and conservation, including through future ‘public money for public goods’ funding models.

#### Ecological

All visions incorporated nature recovery, with conservationists emphasising resilience and connectivity: *‘it’s building up that ecological network so that you’ve got resilience’ (Conservation, Elenydd)*. Conservation, Land and some Access stakeholders described transformative changes such as reintroducing species and enhancing natural flood management, whereas Farmers focused on protecting existing features. Ecological values were linked to wider social-cultural values, such as a strong cultural attachment to locally unique flora. Land stakeholders in the Elenydd emphasised biodiversity value over carbon services *‘we’re doing it for biodiversity, and carbon sequestration is the by-product’*, a sentiment echoed in both landscapes.

In Elenydd all stakeholders, beside Farmers, selected woodland expansion via connecting existing woodlands as a future land-use preference. Land stakeholders in both landscapes highlighted the ecological value of woodlands, as a habitat for Black Grouse in the NPD and for supporting woodland ground flora in the Elenydd. However, all local stakeholder groups raised concerns that future treescape expansion may negatively impact habitats of ecological importance for upland wading birds; ‘*Farming up in the higher farms without the Curlew coming in the Spring and all the Lapwings, Redshank, Golden Plovers and Oystercatchers would be a very different thing, it wouldn’t be nearly as attractive’ (Farmers, NPD)*.

All stakeholders expressed a desire to restore ecosystem functions, allowing natural processes to determine outcomes: *‘It’s working with the environment that we have, you know not trying to change it but to utilise it properly’ (Land, NPD)*. For Farmers, allowing natural processes is about not fighting against nature: *‘the older you get you realise that you’re not as strong as nature, so you plan more and more with it’ (Farmer, NPD).*

Current approaches to treescape expansion were seen as too heavy-handed, with some stakeholders preferring natural colonisation for woodland creation *‘my biggest worry is that we’re putting all this money into planting trees at the moment, but it’s not sustainable and there’s no natural regen coming’ (Access, NPD)*. Stakeholders in the Elenydd emphasised their preferences for natural colonisation, primarily in relation to ecological benefits, but also to preserve landscape heritage via a slower mechanism of change.

#### Social

Land, Farming and Access stakeholders in NPD and Access stakeholders in the Elenydd specified social benefits within their final vision; i.e. promoting future sustainable communities and encouraging a diverse society to care for nature. All stakeholders in NPD described future vibrant communities, local facilities and safeguarding the role of people. This was only captured by Access and Conservation stakeholders in the Elenydd, where Farmers expressed current tensions with the wider community – ‘*people who live in towns, they’re quite happy not to see a farmer’* – describing how this may be exacerbated in the future as the younger generation of farmers move into other careers driven by the financial challenges of farming.

All stakeholders, particularly Farmers and Land, felt people have a key role to play in managing future landscapes, ‘*It’s the people who [are] at the heart of it and if you take them out of the equation a great number of things will change and not necessarily for the better’ (Farmer, NPD)*. Conservation stakeholders in NPD challenged assumptions of negative social values associated with landscape restoration projects; *‘We don’t want to depopulate the local landscape. In fact, quite the reverse, we would quite like people to come and live here’.*

Farmers in both landscapes interpreted sustainability as farming the land within its carrying capacity and ‘*with nature’*. Stakeholders recognised that any future forestry industry would need to be environmentally sustainable, but only Land stakeholders incorporated new commercial forestry into their final vision (Supplementary Table S4). Land stakeholders in NPD applied sustainable and economic values to utilise food and timber products locally ‘*All of them also make the local economy much more resilient because one is less dependent on fluctuations in marketplace, it’s more under your own control’.*

Access and Conservation stakeholders placed more emphasis on food and farming values in the Elenydd than in the NPD, with Access stakeholders in the Elenydd noting *‘I’d like to see farmers get paid for growing what we want, to feed the population’*. Farmers in the Elenydd and both Farmers and Land stakeholders in NPD, specified future food production in their final vision, connecting this to other services: *‘Resilient food production needs to fit in with nature and the environment’ (Farmer, NPD)*. Land-use changes underpinned by food and farming values include trees as shelter for livestock, improving soil health, and a general sense that what is good for nature is good for livestock. Farmers felt wood pasture and hedgerows could benefit farm systems if this did not compromise grass productivity.

All stakeholders emphasised the importance of interconnected regulatory services primarily through flood, water and carbon regulation. Stakeholders’ selection of species-rich grasslands and peatland restoration as future land-use changes were associated with the services they could provide, with only some mention of trees regarding their potential for water and carbon storage *‘It’s scrub in order to meet the hydrology objectives’ (Conservation, NPD).*

Access stakeholders focused most on access and recreation values, especially in NPD. Topics included making the landscape more accessible and encouraging people to enjoy nature and the outdoors, noting that increased access must be delivered sustainably. Future tourism was linked to economic benefits, however overarchingly stakeholders felt access and recreation were more important for local health and wellbeing benefits. In the Elenydd, whilst Land stakeholders recognised that increasing future tourism were part of their organisational strategy, it conflicted with their own personal values and visions for the landscape. Land stakeholders in the Elenydd used phases such as ‘*quiet enjoyment’* to illustrate their preference over financially motivated tourism ‘*I’ve had to fight previous managers who’ve come in, who [have] worked for things like Alton Towers and all sorts … Actually, that’s not what we’re about’.*

#### Cultural

Cultural values were deeply expressed by Farming and Access stakeholders, detailing how culture and heritage underpin the landscape’s fabric, wishing to protect heritage features and ensure the future for people within this landscape *‘if you look at your heritage and your history it’s like a skeleton. Your whole community hangs from that skeleton and gives it flesh’ (Farmer, Elenydd)*.

Both landscapes have a history of upland hill farming; ‘*our landscape has been managed in this kind of way for – what  −  800 years?’ (Farmers, NPD)*. In the Elenydd, Farmers reflected on old droving practices, moving large groups of livestock across the landscape, and the traditional use of horses in land management. Farmers felt this landscape character was symbolic of their own identity. Access stakeholders in both landscapes often shared stories of local histories during discussions, both through farming and other traditional industries.

Heritage values were represented in future land-use preferences as a desire to protect boundary features, veteran trees and field patterns which collectively make up the landscape’s character. Access stakeholders in both landscapes also included protecting archaeological sites and mining features from land-use change. Access and Conservation stakeholders in the Elenydd expressed preferences for using local tree varieties in future tree planting activities given their cultural significance.

In the Elenydd, the compulsory land purchase and subsequent flooding of the valley has had a lasting effect on the community, including the loss of three manor houses, 18 farms, a school and an iconic church which was later rebuilt. Local people still feel anger and hurt *‘[There is a] history of water imperialism. Birmingham saying, “We’re telling you we want your water and you’re going to change the landscape”’ (Access, Elenydd)*. The loss of in-bye land (enclosed pasture typically situated closest to farm buildings) has also resulted in more intensive moorland grazing. Access stakeholders in the Elenydd spoke negatively of the dams’ connections to slave histories: ‘*the money that paid for this was all money from slavery because that’s where Birmingham got its wealth’*. Conversely, Land stakeholders described how today more water is retained in Wales than sent outside the country. They also believed locals feel pride in the dams’ role as a testing site for the famous dam busters during WW2 *‘they might not have felt like they did a lot, but that series changed the war’ (Land, Elenydd)*. In the Elenydd future visions were shaped by history, both physically, and through local stakeholders’ relationship with how changes have taken place through history.

Farmers in both landscapes felt a sense of duty for the management of these landscape and felt their role as stewards should be protected; *‘you take farming out of the equation you’re not then going to have those species of meadows because there would be nobody to maintain or to cut them’ (Farmer, NPD)*. Farmers were proud of the ecological value of the farmed landscape ‘*we’ve got the best wildlife in the country’ (Farmer, NPD)*. Farmers also expressed how their role as stewards contributes to the culture of the landscape by adding character to the countryside.

Farmers felt strongly about protecting their way of life and the landscape for future generations: ‘*We actually love where we are, we’re not here to pollute it, we’re not here to damage it, we want it for our next generations’ (Farmer, Elenydd)*. Stewardship values were expressed by Farmers as a deep pride for the physicality of their jobs and the health and biodiversity value of the food they produced: ‘*I love what our land was able to do and produce, the nutritional value of it is absolutely amazing. Every single bit of biodiversity, it’s all in, that is the story of the landscape’ (Farmer, Elenydd)*. Farming stakeholders in both landscapes felt undervalued in their stewardship role: ‘*If you take the farmers out of the area it would change very, very dramatically and probably not in the way that people want, unless you want to see the whole areas filled with mature trees in the valleys*’ *(Farmer, NPD)*. Stewardship values were not expressed by all stakeholders, despite all groups valuing the services being delivered through land management.

Aesthetic values were associated with treescapes, the value of light and airy woodlands, boundary features and woodland ground flora. However, stakeholders also felt future treescape expansion may threaten aesthetic values, particularly the open moorland vista. Land stakeholders in NPD noted examples where estates have planted large numbers of trees in a considerate way with minimal visual impact on the landscape. In the Elenydd all stakeholders were happy to accept visible scrub and scattered trees across moorlands and outside of small upland valleys. This habitat mosaic is termed Ffridd and is considered culturally important in Wales. Commercial forestry, particularly clear-felling practices, had a negative aesthetic value, described as ‘unnatural’. In the Elenydd, Land stakeholders noted that evergreen forestry and large conifer trees were valued by visitors. When describing old photos of the Elenydd, Access stakeholders wanted to protect this cultural aesthetic from a time they themselves haven’t lived through: ‘*I’ve got a good collection of the Victorian postcards, the black and white ones of about 100 years or more ago and it’s great to look at these. In fact, there’s hardly any trees then’.* Access stakeholders also emphasised a preference for natural colonisation rather than more interventionist tree-planting options because of the negative visual and environmental impact of plastic tree planting tubes.

All stakeholders described the beauty of their respective landscapes through aesthetic values. However, conservation stakeholders in NPD tended to relate this value to visitors, with themselves instead feeling distressed by the landscape’s visual aesthetic, attributing this to their awareness of ecological degradation: *‘I think we do see the landscape in such a different way to a lot of people and it is quite … It’s harrowing. I drive around with my mum and she’s like, “Oh the moorland is beautiful isn’t it”. And I’m just dying’ (Conservation, NPD).*

### Linkage between contextual factors and land-use preferences

Seven socio-political and biophysical contextual factors were referenced by stakeholders as they created their future vision (Table 4). We explore how these contextual factors were considered by different interest groups and infer how they shape future land-use preferences.

**Table 4.** Definitions of contextual factor themes influencing land-use preferences.

Contextual factors	Description
**Socio-political context**	The social or political factors relating to how the environment is managed, either through financing, policies or legislative regulations.
Statutory context	Emphasis placed on the statutory context, and its influence on land-use change such as designations, protected status, regulations and legislations.
Policy context	Policy decisions implemented at a national or regional level which shape land-use change, primarily in relation to net zero or agri-environmental policy.
Financial instruments	Land-use change considered through the availability of different financial instruments.
Reputational	The shaping of decisions by the reputational image presented by, or obligations of, an organisation.
Technology and innovation	Changes facilitated by new ideas, technology and approaches.
**Biophysical context**	Environmental and anthropogenic factors relating to how the environment is managed.
Land management activities	Alterations to how the land is managed and the potential options different actions present.
Climate change	Future uncertainty directed towards the context of a rapidly changing climate, which will influence land-use changes.

#### Statutory context

Both landscapes contain large proportions of ecologically designated areas, i.e. National Nature Reserves, Site of Species Scientific Interests, Special Areas of Conservation and, in NPD, a National Landscape and National Park. All groups specified protecting priority habitats and species in their future vision (Supplementary Table S4) and were concerned that treescape expansion could threaten existing ecological and service values: *‘You could plant trees anywhere you wanted in the country; you couldn’t create a head of moorland anywhere you wanted in the country’ (Farmers, NPD).*

Designations were, however, felt to be too restrictive on management choices and inflexible to trying new approaches such as, low-density planting on grasslands or grazing within woodlands. However, Conservation stakeholders in NPD noted a desire to see more legal protection for veteran trees and wildlife, despite some of the inflexibility that designations create. Farmers felt future land management decisions should be left to the local people who know the land best rather than being prescribed through designations.

Both upland landscapes have significant areas of common land (land jointly shared by multiple individuals and managed under common rights), which stakeholders described as restricting both treescape expansion and management change. Whilst tree planting on common land wasn’t a preference for stakeholders, common laws did limit ambitions around natural colonisation due to statutory barriers to grazing exclusion (Supplementary Table S4).

Both landscapes are shaped by water catchment status, particularly in the Elenydd where stakeholders referred to grazing, fencing and management restrictions near the reservoir-edge. Land and Conservation stakeholders were keen to overcome statutory barriers to allow future grazing of cattle in woodlands around the reservoir for ecological benefits.

#### Policy context

Stakeholders consistently expressed a sense of duty to sequester carbon, stating the significance of these landscapes’ peatland habitats to a national context. Farmers in the Elenydd recognised a need to learn and change future moorland management practices in reflection of Welsh policy surrounding net zero targets *‘I’m quite interested in this peat thing, moving forward and thinking about how we can be good farmers*’.

Stakeholders did feel a responsibility towards national timber security, but this was not included in their land-use preferences as these local areas were deemed not suitable for timber production due to quality of the land: *‘By 2050 we are going to need to up our production of timber in this country if we’re going to have sustainable building of housing’ (Farmer, NPD)*. Stakeholders preferred timber production for local use rather than for national timber security. Land and Farming stakeholders favoured strengthening local trade loops, akin to this more localised worldview; *‘a lot of it is keeping control of what our natural resources are and localising stuff’ (Land, NPD).*

The England Trees Action Plan 2021–2024 (Defra 2021) sets out planting targets of 30,000 ha per year by 2025 whereas the Woodlands for Wales Strategy stipulates a minimum tree planting target of 2,000 ha each year from 2020 (Welsh Government 2018). Whilst stakeholders supported some treescape expansion, most felt these targets where unsuitable for upland landscapes and national agendas did not change their own land-use preferences. The current mechanisms available for delivering these targets (i.e. woodland creation grants) were felt to be inconsiderate of local context and inflexible to allow for their preferred low-density planting and natural colonisation, considered more suitable to the landscapes character and cultural values.

Stakeholders in both landscapes believed government support, namely through agri-environmental schemes, is required to support land-use changes, primarily through financial incentives. England and Wales are currently transitioning towards ‘public money for public goods’ land management schemes. However, Farmers felt sceptical of the shifting nature of agri-environment policy in both England and Wales, describing new policies as ‘*following trends’*. Tenant farmers felt financially reliant on agri-environment payments, compounded by the phasing out of Single Farm Payment scheme. Conservation stakeholders in the Elenydd attributed the current lack of willingness by farmers to plant trees or graze within woodlands to the outgoing area-based Single Farm Payment scheme where trees were deducted from the claimable area for payment. Further flexibility in woodland grant options, and the consideration of ongoing woodland management, could support woodland creation on farms with suitable livestock grazing to improve woodland conditions. Conservation stakeholders raised a lack of policy level support for nature recovery limiting ability to enact their future land-use preferences, *‘How do we even begin to think about this with the current political situation? I know we’ve got a general election coming up, but it’s just I just can’t see us getting any support for it at all’* and a need for more joined-up land-use policy between people, place, nature and ecosystem services.

#### Financing instruments

All stakeholders discussed future payments for public goods in line with current shifts in UK agri-environment policy. However, land stakeholders in NPD and Farmers in both landscapes included food production in their final vision to emphasise that payments for public goods should support, not replace food production *‘how could that change to support wider ecosystem services but seen through the lens of food production*’ *(Land, NPD)*. Farmers felt reliant on funding incentives to make farming profitable, and felt restricted in their future land-use decisions by what agri-environmental schemes could offer financially: *‘they’re just waiting on the next Welsh government scheme in terms of funding’ (Land, Elenydd)*, and felt increasing uncertainty as to the stability and direction of what future schemes will offer under the UK’s agricultural transition. This was particularly prominent for tenant farmers who felt the pressures of meeting tenancy payments.

#### Technology and innovation

Novel technological solutions were referred to by stakeholders in NPD, in particular Land stakeholders. Topics ranged from agricultural practices to research and development, water management, electrification, and mining. Farming and Land stakeholders in NPD related ecotourism management as an innovation opportunity for landowners.

#### Reputational

In NPD Farming and Access stakeholders raised concerns that organisations and local authorities were engaged in tree planting as a tick box exercise without proper consideration: *‘you’ve got to be seen to be doing things, whether or not it’s the right thing or not’ (Access, NPD)*. Land stakeholders in the Elenydd saw tree planting as an offsetting opportunity against their organisational carbon footprint.

#### Management activities

Stakeholders expressed preferences for different management approaches, despite valuing the same ES outputs. For example, to reduce wildfire risk in NPD Land stakeholders focused on livestock management, whereas Conservation stakeholders emphasised restoring hydrological functions. Local stakeholders described different preferences for future grazing management, from rotational systems to reduced or maintaining moorland grazing, whilst all seeking similar outcomes of improved soil health, biodiversity value and fire risk. Conservation stakeholders focused less on maintaining status-quo and more on reverting unsustainable land management practices, in NPD through management for driven grouse shooting and in the Elenydd through overgrazing. Whilst some Farmers were actively trying out new land management approaches already, others felt strongly against future land management changes, believing traditional management was most appropriate given the benefit of knowledge having been passed down through generations of farming on the land.

All groups described a desire for more partnership working and joined-up management. By taking a holistic approach, land-use preferences focused on transitional habitats for improving connectivity and softening edges between different land-use types. Scrub was frequently identified to fulfil this purpose (Supplementary Table S4).

#### Climate change

Local stakeholder decision-making about future land-use is placed in the context of future climate change, both in terms of climate adaptation and through the uncertainty of outcomes in a changing system. *‘I think it’s fairly aspirational, the 2050 vision, to actually be able to maintain what we’ve got, I think we’ll struggle to do that with climate change’ (Farmer, NPD)*. The major focus in the Elenydd was around future flood and drought adaptation to develop a more climate aware community. In NPD additional themes included disease risk, wildfires, shifting species ranges and the need for adaptive management.

Peatland restoration was a priority for all stakeholders for mitigating future climate change with a sense of pride expressed in this being a special upland landscape. Little value was attributed to treescape for their carbon storage potential outside of the Conservation stakeholder group, with some believing tree growth would not be sufficient at altitude. However, all stakeholders discussed on-farm treescape expansion to benefit livestock under future climate change, despite not all expressing food and farming priorities. Farmers in NPD used climate change to emphasise the importance of their future role in food production: *‘with climate change there’s going to be vast areas of the world that will not be producing food and it will be very short sighted not to be producing food up here’.*

## Discussion

In this study, we apply Fedele’s framework (Fedele et al. 2018) to conceptualise the linkages between stakeholder values, context and future land-use preferences through a deliberative PSP approach. Our study generates new empirical data comparing stakeholder values and land-use preferences for treescape expansion in two UK upland landscapes. Understanding the complex nuances of values and context requires space for deliberative unpacking of land-use preferences. The discussion explores the resulting opportunities and challenges for treescape expansion at local scales through three lenses; treescapes as a mechanism of delivering ES, underlying values which align with treescape expansion and the influence of wider context on land-use preferences.

### Treescapes and the delivery of future ecosystem services

When creating future land-use visions, stakeholders struggled to rank coarse land uses, instead preferring a mosaic of habitats at both a landscape and management level. Stakeholders felt the landscape should, and could, deliver multiple interlinked ESs, connecting biodiversity, carbon and water services. Whilst this is akin to existing narratives of multifunctional landscapes of treescape scenarios (Burton et al. 2019) and mirrors policy-shifts towards achieving win-win solutions for delivering across multiple outcomes, there is also growing recognition of the trade-offs associated (The Royal Society 2023; UK Parliament 2024). Very few groups (only Farming and Land stakeholders in NPD and Farming only in Elenydd) included food production as a priority service, complementing land-use scenario literature demonstrating food production is often trade-off to achieving multiple environmental benefits (Finch et al. 2021). Conservation and Land stakeholders in the Elenydd emphasised regulatory services, with livestock as a tool to deliver this, but did not place the same emphasis directly on food production as farmers, or Land stakeholders in NPD. In our study ES benefits, in particular carbon sequestration, were more typically expressed through other land uses, namely peatland and grassland, rather than attributing additional benefits through future treescapes (Sing et al. 2018), with the exception of biodiversity and flood risk management.

Our approach considers future treescapes in combination with wider land-use as part of a deliberative visioning exercise. This approach is particularly relevant for understanding treescape opportunities within UK uplands, where treescape expansion is met with concerns of impacts on existing management of peatland and grassland habitats. Whilst existing approaches, such as Q-method, have particular strengths in synthesising the breadth of attitudes towards treescape expansion (Urquhart et al. 2012; Iversen et al. 2022), a more deliberative approach avoids creating broad typologies, when in fact multiple interlinked values and contextual factors may underpin resulting preferences. In addition, PSP creates space for contradictory values in relation to land-use to be realised; that may often be unaccounted for in more structured value assessments (Duesberg et al. 2013).

The UK evidence base for woodlands typically focuses on biodiversity and regulating services, but evidence gaps remain around services from wider forms of treescapes besides plantations (Burton et al. 2018). Whilst the concept of multifunctional woodlands is well-established (Paletto et al. 2012), limits are enforced by the biophysical boundaries of ecosystems, ultimately forcing trade-offs between different land-use choices (Goldstein et al. 2012). Stakeholders’ reluctance to rank land-use preferences not only re-enforces the need for understanding diverse values and preferences, but raises questions over how trade-offs are conceptualised during decision-making.

Whilst all stakeholders found opportunities for increasing tree cover, many of the spatial criteria around tree planting were influenced by the perceived risk of trees to existing ESs, including heritage and aesthetic values, food provisioning, carbon sequestration, and wading birds. Our findings agree with existing literature on upland treescape expansion, documenting stakeholder concerns to existing landscape features and the pressures of national planting targets (Fitzgerald et al. 2021).

Regarding aesthetic values, treescapes were felt to negatively impact open moorland characteristics. However, other studies in the NPD have claimed that up to a 75% woodland cover scenario would not incur a trade-off with the aesthetic requirements of nature-based recreational tourism (Iversen et al. 2023). Similarly, other studies of treescapes in upland landscapes have shown that stakeholders express cultural benefits of treescapes, particularly in relation to tourism and recreation (Fitzgerald et al. 2021). In contrast, our findings show that stakeholders connect values associated with treescapes to wider concerns of growing visitor pressure. Cultural values were instead attributed to other landscape characteristics, particularly traditional farming histories, rather than to treescapes. Our findings highlight a difference in the perception of treescapes by local stakeholders who may attribute deeper cultural connections to aesthetic values beyond its economic assessment of tourism revenue.

In this study, conservation stakeholders, as well as some Land stakeholders in the NPD, offered more details around dynamic and transformative landscape changes, akin to existing ‘wild woodland’ scenarios which prioritises woodlands for nature (Burton et al. 2019). However, even in these instances, discussions frequently returned to ensuring the future presence of people within these landscapes and finding ways to integrate social and ecological benefits.

### Unpacking values uncovers treescape opportunities

When stakeholders had the space to unpack broad land uses changes and create their own criteria for land-use change; opportunities for expanding tree cover in upland landscapes emerge. Stakeholders express a wide range of ecological, economic and socio-cultural values; and within this value space, nuanced opportunities exist (Breyne et al. 2021). This supports the findings of other values-based approaches to treescape scenario design that show stakeholder values allow for a greater increase in tree cover in uplands than anticipated (Fitzgerald et al. 2021). Stakeholder treescape preferences; non-woodland treescapes such as scrub and scattered trees, conversion of conifers to broadleaf, connected existing woodland and converted unproductive land, aligned most with stakeholders’ values of landscape. Whereas woodland creation, large-scale tree planting and high-altitude woodland expansion were less compatible with local values and preferences. For example, in the Elenydd, stakeholders were in favour of natural colonisation of Ffridd habitat (the area between enclosed fields and open moorland characterised by heather, bracken and scattered trees), recognising this as a culturally significant habitat type and creates more subtle changes to the landscape. Focusing on natural processes for treescape expansion can prove more sympathetic to the landscape’s character than tree planting (Bowditch et al. 2023). In other UK upland case-studies, treescape opportunities favour this more natural looking and scattered approach (Fitzgerald et al. 2021), especially around river valleys.

Future visions incorporated the role of people within these landscapes, from creating a sense of community to employment and health and wellbeing benefits. For example, future food production was described by farmers as more than the physical product, but linked to the historical role of people within the landscape, their identities as stewards and their role in creating landscape character. Whilst the cultural farming identity resonated with some local community members through histories of the landscape, not all stakeholders expressed this same connection, and therefore, many did not retain food production in their final visions. Changing farming practices often have wider implication to changes in rural communities, with social and cultural connections to employment, local services and rural identity (Murphy et al. 2022). The future role of food production represents deeper cultural connections to place which farmers feel is threatened by land-use change synonymous with restoration visions. For farmers in our upland study regions, certain contextual vulnerabilities they perceive as negatively impacting their livelihoods, identity, and surroundings may shape contemporary narratives of resistance to treescape expansion. Growing social conflicts are evidenced around upland land-use change in relation to existing cultural framings of landscapes (Wynne-Jones et al. 2018). These findings highlight a need to understand vulnerability contexts that may generate social conflicts surrounding land-use change before they occur; echoing similar findings (Vasile 2018) exploring the shaping of pro or anti narratives towards European re-wildling initiatives.

However, whilst farmers raised concerns over woodland creation reducing grass production, when considered in the context of climate change trees became part of their farm resilience solution, via wood pasture, hedgerows and shelter trees. Previous case studies have suggested that climate change is too remote a concept to directly influence farmers during woodland creation scenario exercises (Fitzgerald et al. 2021), but here we see both farmers and other stakeholders shaping their land-use preferences to maximise climate resilience of farm systems through treescapes (Wreford and Topp 2020). Climate adaptation in the uplands is dependent on the function and services of the landscape and relies on the multifunctional combination of services across spatial scales (Richards et al. 2023). This resonated with the preferences expressed within our case studies of the integration of trees through a holistic and catchment-level approach to maximise ecosystem resilience. Stakeholders placed greater consideration on those contexts, such as climate change, which are felt more closely through the regularity in which they engage with their surrounding environment. Whilst climate adaptations in farming was regularly discussed, future forestry was only discussed from an ecological sustainability perspective, with climate context not creating links to expressing climate-resilient forestry practices. Furthermore, carbon storage priorities were associated with peatland restoration rather than tree planting, a reflection of both the perceived lack of suitability for tree growth in uplands, as well as cultural values linked to retaining the traditional moorland aesthetic of upland habitats.

In some cases, different stakeholder values resulted in similar land-use preferences. For example, while most stakeholders attributed peatland restoration to its carbon sequestration and regulatory services, others also included its aesthetic and cultural values in their reasoning. However, we also identified cases where stakeholders shared the same land-use preferences, but wanted it achieved through different mechanisms, primarily regarding grassland management approaches for achieving regulatory ESs. Management choices were linked to stewardship values of farmers, and the importance of traditional practices. Whilst culturally embedded practices can change, for example farmers in the Elenydd described their shifting perspective on peat extraction after gaining an awareness of the carbon value of peat, when it came to the role of livestock these preferences were deeply embedded within their culture. Management approaches to woodlands have different perceived ES impacts (Eastwood et al. 2024). These subtleties are often not captured in long-term transformative scenario-creation methodologies. Our approach using values, contextual factors and land-use preferences can bring together insights on both abstract long-range visioning preferences as well as capturing subtleties of incremental management choices.

### Treescape decision-making within landscape-specific contexts

By considering the role of contextual factors within decision-making processes (Fedele et al. 2018), more nuanced insights are generated on land-use preferences. Stakeholder values are embedded within the places in which they are situated, evident through contrasts in preferences between similar stakeholders across the two landscapes. Therefore, when designing treescape expansion, local stakeholder preferences must be captured under different place-based contexts to combine sense of place and ES theory in future ES valuation approaches (Gottwald et al. 2022). Food and farming values were discussed by Farmer and Land stakeholders in the NPD, but were not considered by Land stakeholders in the Elenydd. Land stakeholders in the NPD consist of estate owners with a history of food production namely in game meats, whereas Land stakeholders in the Elenydd were primarily water managers and don’t necessarily share this historical connection to food and farming. The historical context of these landscapes, and their past land uses, are shaping current-day values.

Apparent negative attitude of farmers towards tree planting may be embedded in their experiences with top-down policy implementation due to poor consultation processes from government agencies, rather than negative values to trees themselves (Iversen et al. 2022). In our study, policies were perceived negatively as ‘trends’ and ‘fashions’. Economic values acted in combination with sustainable and people values to influence land use preferences not through the profitability of land-use changes but in the availability of funding schemes to support local livelihoods. Therefore, future policy mechanisms should incorporate training, knowledge exchange and smaller-scale integrated options for treescapes with greater flexibility to compliment local stakeholder preferences.

## Conclusion

The paper aligns with recent approaches to sustainability that understand societal values, preferences and behaviour as enculturated, that is, as co-evolving in socio-cultural and biophysical contexts (Schill et al. 2019). This supports a more comprehensive understanding of stakeholder values, with growing international and interdisciplinary literature placing greater emphasis on stakeholder perspectives within spatial scenario modelling, land-use decision-making and multifunctional land-use frameworks (Kariuki et al. 2021; Zscheischler 2021; Harmáčková et al. 2022; Lin et al. 2024). While our paper addresses this, connecting our study to Fedele’s contextual analysis enhances understanding of how cultural systems act in competition with other structural factors to shape a community’s land-use and decision-making preferences. This, in turn, contributes to a more nuanced understanding of the factors that shape the socio-political acceptability of land-use change. Without these deliberative processes, preferences may be oversimplified, and local actors alienated from decision making. Whilst this paper has applied this to identify opportunities for treescape expansion, this approach can be replicated across land-use planning issues. This could be furthered still by connecting values, contexts and preferences to actions and outcomes, completing Fedele’s cycle (Fedele et al. 2018).

Opportunities emerged for treescape expansion, such as through culturally-sensitive low density natural colonisation. Treescapes, can offer multiple benefits from the perspective of local stakeholders, primarily around water storage, climate and biodiversity, however overarching concerns remain that tree planting threatens upland habitats and the ES they provide. Whilst all stakeholders identified opportunities for increasing tree cover, scaling up from the landscape-scale to deliver national objectives requires flexibility in the application of treescape policy to align with the depth of socio-cultural values shaping land-use preferences.

## Supplementary Material

Supplementary material.docx
