# Supplementary material for: Visioning future treescapes in upland landscapes: using deliberative processes to understand values and land-use preferences of local stakeholders
Source: Ecosyst People (Abingdon). 2025 May 9;21(1):2497823. doi: 10.1080/26395916.2025.2497823 (PMC12312000; doi:10.1080/26395916.2025.2497823)
Supplement: Supplementary material.docx [file TBSM_A_2497823_SM0305.docx]

**Supplementary Material**

Table 1: Values thematic analysis, breakdown of sub-codes by stakeholder type and landscape and coding frequency. Farming focus group indicates as Y=yes, N=No for presence/absence.

|  | North Pennies and Dales | | | | Elenydd | | | | |
| --- | --- | --- | --- | --- | --- | --- | --- | --- | --- |
|  | Land | Access | Conservation | Farming | Land | Access | Conservation | Farming Focus Groups |  |
| Economic |  |  |  |  |  |  |  |  |  |
| Financing for public goods |  | X | X | X |  | X | X |  |  |
| Creating jobs through land management and restoration |  | X | X |  | X |  | X |  |  |
| Need to make hill farming profitable |  |  |  |  | X | X | X |  |  |
| Stewardship schemes as a solution for biodiversity | X | X |  |  |  |  |  |  |  |
| Livestock breed selection based on price |  |  |  | X |  |  |  |  |  |
| Create an economic return for predator control |  |  |  | X |  |  |  |  |  |
| Future farming as ecotourism diversification | X |  |  | X |  |  |  |  |  |
| Tourism benefits to the future local economy | X |  | X |  |  | X |  |  |  |
| Following funding incentives | X |  |  | X |  |  |  |  |  |
| Access as a public goods payment |  | X |  |  |  |  |  |  |  |
| Economic support to farmers |  | X |  | X |  |  |  |  |  |
| timber industry provides capital for biodiversity projects |  |  |  |  | X |  |  |  |  |
| Need for investment |  |  |  |  | X |  |  |  |  |
| Economic value of forestry |  |  |  |  |  | X |  |  |  |
| Social |  |  |  |  |  |  |  |  |  |
| People and Community |  |  |  |  |  |  |  |  |  |
| Secure future businesses and support rural livelihoods | X |  | X |  |  | X |  |  |  |
| Provide training to land managers to deliver restoration |  | X | X |  |  |  | X |  |  |
| Communities and facilities |  | X | X | X |  |  |  |  |  |
| Opportunities for young people | X |  |  |  |  | X |  |  |  |
| Creating vibrant communities |  | X | X |  | X | X | X |  |  |
| Products should be for local people | X |  |  | X |  |  | X |  |  |
| Community engagement |  | X |  | X |  | X |  |  |  |
| Future health and wellbeing | X |  |  | X | X | X |  |  |  |
| People are valued as a natural resource | X |  |  |  |  |  |  |  |  |
| Future working landscape |  |  |  | X |  |  |  |  |  |
| Healthy food for people |  | X |  | X |  |  |  | X |  |
| Increasing diversity of people |  | X |  |  |  |  |  |  |  |
| Stronger tenancies and power to farmers |  |  |  | X |  |  |  |  |  |
| Devolution of decision making to local people | X |  |  | X |  |  |  |  |  |
| Keeping people in the landscape | X | X | X | X |  |  |  |  |  |
| Local currency system |  |  |  |  |  |  | X |  |  |
| Local prosperity |  |  |  |  |  | X |  |  |  |
| Regeneration of people and landscape |  |  |  |  |  |  |  | X |  |
| Sustainability |  |  |  |  |  |  |  |  |  |
| Drive to be sustainable | X | X | X | X | X | X | X |  |  |
| Future resilience | X |  | X | X |  |  | X |  |  |
| Future green energy | X | X | X | X | X |  | X |  |  |
| Designing biodegradable tree guards (no plastics) | X |  |  |  |  |  | X |  |  |
| Reduce carbon emissions from industry | X | X | X |  |  |  |  |  |  |
| Low carbon farming | X | X | X | X |  |  |  |  |  |
| Chemical free farming | X |  | X |  |  |  |  |  |  |
| Sustainable communities |  |  | X | X |  |  |  |  |  |
| Re-purposing old buildings | X |  |  |  |  |  |  |  |  |
| Sustainable farm businesses |  |  |  |  | X |  |  |  |  |
| Local sustainability in circular economies |  |  |  |  |  | X | X |  |  |
| Future sustainable tourism |  |  |  |  |  | X |  |  |  |
| Sustainable forestry |  |  |  |  | X | X | X |  |  |
| Farming within the lands carrying capacity |  |  |  |  |  |  |  | X |  |
| Food and Farming |  |  |  |  |  |  |  |  |  |
| Future food production and other animal products | X |  |  | X | X | X | X | X |  |
| Positive farming impacts of wood pasture and agroforestry | X | X | X | X | X | X | X | X |  |
| Maintain more grazing pasture than woodland |  |  |  | X |  |  |  |  |  |
| Biodiversity benefits farmers |  |  |  |  |  | X |  |  |  |
| Interventions on areas not used by livestock (protecting in-bye) |  |  |  | X |  |  | X |  |  |
| Changes only to land doing nothing | X | X |  | X |  |  |  |  |  |
| Meadows should function for farming |  |  |  |  |  | X |  |  |  |
| Services |  |  |  |  |  |  |  |  |  |
| Water services | X | X | X | X | X | X | X |  |  |
| Services provided by trees |  |  |  |  | X |  |  |  |  |
| Carbon services | X | X | X | X | X | X | X |  |  |
| Interconnected services | X |  | X |  | X |  |  |  |  |
| Supporting pollinators | X |  |  |  |  |  |  |  |  |
| Access and recreation |  |  |  |  |  |  |  |  |  |
| A more accessible landscape |  | X | X |  | X | X |  |  |  |
| Recreation a key part of future economy |  | X | X |  | X |  |  |  |  |
| Encouraging more people to enjoy the landscapes |  | X |  |  |  | X |  |  |  |
| A richer landscape is better for visitors |  | X |  | X |  |  |  |  |  |
| Access in future woodlands |  | X |  | X |  |  |  |  |  |
| Cultural |  |  |  |  |  |  |  |  |  |
| Stewards |  |  |  |  |  |  |  |  |  |
| Risk to traditional hill farming | X |  |  | X |  |  |  |  |  |
| Moving back to traditional farming practices |  |  |  |  | X | X | X | X |  |
| Farmers creating the landscapes |  |  | X | X | X | X |  |  |  |
| Empowering farmers to make decision on the land | X |  |  |  |  |  |  |  |  |
| Farmers as custodians |  | X |  | X |  |  |  |  |  |
| Farming isn’t pure economics |  |  |  | X |  |  |  |  |  |
| Farm business collaboration | X |  |  |  |  |  |  |  |  |
| Environmental benefits are the by-product of farming |  |  |  | X | X |  |  |  |  |
| Pride in management work |  |  |  | X |  |  |  |  |  |
| Personal dedication to protecting species |  |  |  | X |  |  |  |  |  |
| Local farming history |  |  |  |  |  | X |  |  |  |
| Generational lag of farming practices |  |  |  |  |  |  |  | X |  |
| History and Heritage |  |  |  |  |  |  |  |  |  |
| Using older native breeds to graze- maintaining gene pool |  | X |  |  | X | X |  | X |  |
| Connections to landscape history and importance of heritage | X | X |  | X |  | X | X |  |  |
| Maintain landscape heritage boundary and veteran features |  |  |  | X |  | X | X | X |  |
| Trees threatening the landscapes cultural heritage | X | X |  | X |  |  | X |  |  |
| Concerns over loosing local skills for maintaining heritage features and crafts |  |  |  |  |  | X |  |  |  |
| Creating heritage for the future | X | X |  |  |  |  |  |  |  |
| Importance of people understanding the history of the area | X | X |  |  |  |  |  |  |  |
| Telling stories about the landscape's history |  |  |  |  |  | X |  |  |  |
| Pride in heritage and uniqueness | X |  |  |  |  | X |  |  |  |
| Local heritage of tree varieties |  |  |  |  |  |  | X | X |  |
| Restoration of old buildings |  |  |  |  |  |  | X |  |  |
| History of dam building |  |  |  |  | X | X | X |  |  |
| Local passion for the landscape |  |  |  |  |  |  |  | X |  |
| Aesthetic |  |  |  |  |  |  |  |  |  |
| Beauty | X |  | X |  |  | X |  |  |  |
| Trees having a visual impact on the landscape | X | X |  | X |  | X |  |  |  |
| Countryside aesthetic | X | X |  | X |  | X |  |  |  |
| Negative visual impact of clear felling |  | X |  |  |  |  |  |  |  |
| Visual impact of fencing for tree planting |  |  |  | X |  |  |  |  |  |
| Negative visual impact of green energy | X |  |  |  |  |  |  |  |  |
| Don’t like to see change |  |  |  |  |  | X |  |  |  |
| Aesthetic value of meadows |  |  |  |  |  |  | X |  |  |
| Aesthetic value of the hills |  |  |  |  |  |  |  | X |  |
| Tree planting looks un-natural |  | X |  |  |  |  |  |  |  |
| Ecological |  |  |  |  |  |  |  |  |  |
| Intrinsic value of nature |  | X |  | X | X |  | X | X |  |
| Increased biodiversity | X | X | X |  | X | X | X | X |  |
| Species-reintroduction | X |  | X | X |  | X |  |  |  |
| Lawton principles | X | X | X |  |  |  | X |  |  |
| Protecting ground nesting birds and improving habitat | X | X | X | X | X |  | X |  |  |
| Resilient ecosystems | X |  | X |  |  |  |  |  |  |
| Protecting and restoring important habitats (including protecting from tree planting) | X | X | X | X |  | X | X |  |  |
| Trees for biodiversity not timber | X |  |  |  |  |  |  | X |  |
| Protect wildlife from impact of grouse management |  | X | X |  |  |  |  |  |  |
| Protect ancient woodlands |  | X |  |  |  |  |  | X |  |
| Any changes to be sympathetic to the environment | X |  |  |  |  |  |  |  |  |
| Securing sensitive sites | X |  |  |  |  |  |  |  |  |
| Pride in wildlife |  | X |  | X |  |  |  |  |  |
| Rare lichens on ash trees |  |  |  |  | X |  | X |  |  |
| Benefits of trees for nature | X |  |  | X |  |  |  | X |  |
| Keeping people out the landscape |  |  | X |  |  |  |  |  |  |
| Allowing natural processes | X | X | X |  | X | X | X |  |  |
| Focusing on natural colonisation | X | X | X |  | X | X | X |  |  |
| Working with nature | X |  |  | X |  |  | X |  |  |
| Everything is connected | X | X | X | X | X |  | X |  |  |

Table 2: Contextual Factors thematic analysis: break down of sub-codes by stakeholder type and landscape Farming focus group indicates as Y=yes, N=No for presence/absence.

|  | | | | | | | North Pennines and Dales | | | | Elenydd | | | |
| --- | --- | --- | --- | --- | --- | --- | --- | --- | --- | --- | --- | --- | --- | --- |
|  | | | | | | | Land | Access | Conservation | Farming | Land | Access | Conservation | Farmer Focus Group |
|  | **INSTITUTIONAL CONTEXT** | | | | | | | | | | | | | |
| Statutory Context | | | | | | |  |  |  |  |  |  |  |  |
| Influence of designations within the landscape | | | | | | | X | X | X | X | X | X | X |  |
| Common law causes land use change restrictions | | | | | | | X |  | X |  |  | X |  |  |
| Legal protection required for veteran trees | | | | | | |  |  | X |  |  |  |  |  |
| National Landscape status causes planning restrictions | | | | | | | X |  |  | X |  |  |  |  |
| Natural England can be resistant to certain land use changes | | | | | | |  |  | X | X |  |  |  |  |
| Agri-environment schemes don’t allow for natural colonisation approaches | | | | | | |  |  | X |  |  |  |  |  |
| Rules are holding back enabling species re-introduction | | | | | | | X |  |  |  |  |  |  |  |
| Felling licence place restrictions on land use change | | | | | | |  | X |  |  | X |  |  |  |
| A need for less black and white designations | | | | | | |  |  |  | X |  |  |  |  |
| Special species status is upsetting the ecological balance | | | | | | | X | X |  | X |  |  |  |  |
| Water catchment restrictions | | | | | | |  |  |  |  | X | X |  |  |
| Policy Context | | | | | | |  |  |  |  |  |  |  |  |
| Changes to land use policy | | | | | | |  | X | X | X |  | X | X |  |
| Importance of national timber security | | | | | | |  |  |  | X | X | X |  |  |
| Changing land ownership (private to public) | | | | | | |  |  | X |  |  |  |  |  |
| Reliance on top-down action for change | | | | | | | X |  | X |  |  |  |  |  |
| Need for government support | | | | | | | X |  |  |  |  |  |  |  |
| Limited by current uncertainty of political climate | | | | | | |  |  | X |  |  |  |  |  |
| Top-down water policies | | | | | | |  |  |  |  | X | X | X | X |
| Shifting approaches in Welsh tree policy | | | | | | |  |  |  |  | X |  | X |  |
| Financial instruments | | | | | | |  |  |  |  |  |  |  |  |
| Land management trends caused by funding schemes | | | | | | | X | X | X | X | X | X | X | X |
| Future carbon markets and net gain financing | | | | | | | X |  |  |  | X |  | X |  |
| Need for longer term funding schemes | | | | | | |  |  | X |  |  |  |  |  |
| Influence of potential premium product markets | | | | | | | X |  |  |  |  | X | X |  |
| Stewardship funding as a solution to biodiversity | | | | | | | X | X |  |  |  |  |  |  |
| Tenancies place financial restrictions on land management | | | | | | |  |  | X | X |  |  |  | X |
| Risk to farmers from losing basic payment | | | | | | |  |  |  | X |  |  |  |  |
| Need for more investment into the landscape | | | | | | |  |  |  |  | X |  |  |  |
| Creating a market for non-commercial timber species | | | | | | |  |  |  |  |  |  | X |  |
| Technology and Innovation | | | | | | |  |  |  |  |  |  |  |  |
| Land use solutions in technology | | | | | | | X |  | X | X |  |  |  |  |
| Lithium mining | | | | | | | X |  |  |  |  |  |  |  |
| Exploring carbon farming | | | | | | |  |  |  | X |  |  |  |  |
| Farming futures in ecotourism diversification | | | | | | | X |  |  | X |  |  |  |  |
| Research and development activities | | | | | | | X |  |  |  |  |  |  |  |
| Sabre planting | | | | | | |  |  |  |  |  |  | X |  |
| Central slurry management | | | | | | |  |  |  |  |  |  | X |  |
| Reputational | | | | | | |  |  |  |  |  |  |  |  |
| Compliance driven activities | | | | | | | X |  |  |  |  |  |  |  |
| Management decisions based on following the guidelines | | | | | | |  |  | X |  |  |  |  |  |
| Tree planting as a tick boxing action | | | | | | |  | X |  | X |  |  |  |  |
| Organisational net zero obligations | | | | | | |  |  |  |  | X |  |  |  |
|  | |  |  |  |  | **BIOPHYSICAL CONTEXT** | | | | | | | | |
| Management Activities | | | | | | |  |  |  |  |  |  |  |  |
| Emergence of new approaches to farm management (High nature value and regenerative agriculture) | | | | | | | X | X | X |  |  | X | X |  |
| Improved ongoing woodland management | | | | | | | X | X | X |  |  |  | X |  |
| Managing existing areas better/getting the management right | | | | | | | X |  | X | X |  |  | X |  |
| Changes to grazing management- species and density | | | | | | | X | X | X | X | X | X | X | X |
| Partnership and joined up management | | | | | | | X | X | X |  | X | X | X |  |
| Facilitating opportunities to actively try something different | | | | | | |  | X |  |  |  |  |  |  |
| Catchment level, dynamic management | | | | | | | X | X | X |  |  |  |  |  |
| Deer management | | | | | | | X | X | X |  |  |  |  |  |
| More management control to farmers | | | | | | |  |  |  | X |  |  |  |  |
| Need for long term management plans | | | | | | |  |  | X |  |  |  |  |  |
| Need for fencing management to allow natural regeneration | | | | | | |  |  |  |  | X |  |  |  |
| Need for human intervention | | | | | | |  |  |  |  | X |  | X |  |
| Climate change | | | | | | |  |  |  |  |  |  |  |  |
| Climate change impacts and adaptations | | | | | | | X | X | X | X | X | X | X | X |
| Adaptations to flood management | | | | | | | X |  | X | X | X | X |  |  |
| Adaptations to future wildfire risk | | | | | | | X |  | X |  |  |  |  |  |

Table 3: Treescape intervention definitions presented to stakeholders

| **Treescape intervention** | **Definition** |
| --- | --- |
| Semi-natural grassland | Grassland modified by human management but retaining many natural features and species diversity |
| Agroforestry | Tree planting in arable fields (silvoarable) or improved grassland pasture (silvopasture) |
| Wood pasture | Mixed habitat containing individual park like trees |
| Conifer woodland | Evergreen, typically non-native, woodland typically planted for commercial timber production |
| Woodland creation | Woodland creation by planting or natural colonisation near to existing woodland. Native deciduous or mixed deciduous species |
| Alter stocking density *or* alter grazing species | Changes in grazing management on semi-improved or improved grassland by reducing stocking density or switching grazing species (i.e. from sheep to cattle) |
| Low carbon farming | Made up of behavioural and innovative practices to reduce farm carbon emissions |
| Peatland restoration | Restored peatland ecosystem functions |
| Scrub | A mixture of trees and rough grassland |
| Other Trees | Scattered trees on slopes, low density planting, hedgerows or any other form of tree planting. |
| Ffridd* | *Welsh landscape only. The area between enclosed fields and open moorland characterised by heather, bracken and scattered trees |

Table 4a: Future Vision North Pennines and Dales. Number ordering applied where list has been ranked in order of preference by participants.

|  | Vision Statement | Ecosystem Services | Land Use change | Treescape Interventions | Spatial rules |
| --- | --- | --- | --- | --- | --- |
| Access | - Mosaic of habitats - Improved diversity - Carbon sequestration for peatland, grasslands, trees and woodlands - Improving access for all - Increasing education of the natural environment and its evolution - Working in partnership with landowners and communities to reach shared goals - Creating heritage for the future - Encouraging a diverse society to respect, understand and actively care | 1. Biodiversity 2. Carbon sequestration and flood mitigation 3. Recreation and tourism linked to health and well-being benefits 4. Educational opportunities 5. Biocultural heritage | 1. Wood meadows where there are farms below moor line 2. Expand footpaths by increasing networks between settlements and more permissive pathways | 1. Peatland 2. Alter stocking density 3. Scrub   Semi natural grassland Woodland creation  Other trees   1. Wood pasture 2. Low carbon farming | - Peatland only in areas currently used for grouse shooting - More native cattle, less sheep on land currently on open moorland fringes - Only allow scrub on land below open moorland - Woodland creation in the gills, bracken slopes, field margins, to replace conifer plantations and extend for enhanced connectivity - All planting only on marginal unproductive land, with minimal impact on waders - Boundary /field trees can be on any type of land |
| Land | - Local futures- pride in heritage - Carbon and biodiversity friendly farming - Key water management sustainability and natural processes - Working with local resources - More than just farming (business)- farming foundation | - Carbon - Habitat connectivity - Business resilience/economy - Increased biodiversity - Water management - Food production | - Deer management- browsing minimal - Diversifying livestock/ management - Low carbon farming- circular economy/ nil artificial inputs - Renewables- appropriate for environment - Use of technology and future products efficiency - Reservoirs for water storage, security, flow, release, livestock - New tourism infrastructure - Rural business - Floodplain reconnection - Control key invasives - Positioning micro-habitats- connecting species | - Mixed diversifying - Riparian gills, scrub, woodland trees - Meadow expansion - Edge broadleaves - Splitting between upland and lowland - No monocultures - Places for scrub woodland - Fruit bearing trees to support biodiversity - Mosaic (bit of everything) | - Avoid waders - No tree planting on peat - Avoid irreplaceable habitats- calaminarian grassland - Avoid ecologically designated sites |
| Conservation | - Landscape with re-wetted, functional peat, underpinned by scrubby edges - Woodland gills - Valley side accessible woodland - Wood pasture, hedges, and fields tress - Dynamic rivers with full use of floodplains and riparian woodland and scrub | 1. Wildlife 2. Carbon 3. Water 4. Rural livelihoods 5. Recreation and tourism | - Land reform - Deer management - Peatland restoration- hydrology, heathland mosaic, more wooded landscape - High nature value farming incentivised - Regenerative farming - Reduced intensity of grouse moor management - Long term management with proper investment - Outcome focused grants and schemes - Landscape woodland plan - Land use change on commons | - Scrub - Scattered field trees - Valley side woods - Hedges and field trees - Riparian - Montane scrub - Wood pasture - Mixed productive woodland (not just sika) - Planting and natural colonisation | - Prioritise near woodland -connectivity - No planting on peat (but allow natural processes) - Allow planting on steep sided gills and slopes - Avoid priority habitats- but some scattered on heathland - Productive woodlands only on accessible slopes - Waders- use BTO zonal map and adjust sensitivities but allow steep sided/ gill planting- no planting in zone 3 and 4 - Allow a percentage of planting in some wader areas but only if it has wider biodiversity benefits |
| Farming | - Public good as a valuable business income - Maintaining of existing high-quality habitats - Nature and farming together- resilient food production - Sustainable communities and strong local culture with good skills basis - Replace inappropriate conifer woodland - Small scale tree introduction in suitable places that don’t impact existing habitat | 1. Sustainable working landscapes- community driver 2. Food production through good soil health 3. Biodiversity- maintaining high quality habitats within the landscape 4. Water quality, supply and storage | - Maintenance of existing good quality - Restoration of abandoned quarries for nature or water storage - Use unproductive areas for high nature and biodiversity - Small scale renewables at farm or house level | 1. Maintain existing semi-natural grasslands 2. Replace non-economic conifer woodland with broadleaf with possible expansion to link up existing woodlands 3. Scrub and small shrubs or wood pasture/parkland each suitable in different areas- scrub in the gills and wood pasture for livestock shade in lower area 4. Low Carbon farming- on farm renewable energy creations 5. Other trees: replace old standards within hedgerows | - No tree planting on peatland (specifically peatland not blanket bog) - Scrub habitat should only take place in upland gills and not impact the wider landscape - No wood pasture in areas where curlew/waders are found - Priorities wood pasture as buffers around existing woodland - Planting not to introduce on the openness of the landscape and not on wind exposed area - No tree planting on good species rich grasslands and hay meadows |

Table 4b: Future Visions Elenydd; Number ordering has been applied when list has been ranked in order of preference by participants.

|  | Vision Statement | Ecosystem Services | Land Use change | Treescape Interventions | Spatial rules |
| --- | --- | --- | --- | --- | --- |
| Access | - A mosaic of linked, connected habitats for biodiversity - More community engagement for wellbeing, climate change awareness and behaviour change - Unobstructed access for people of all capabilities for the benefit of physical and mental well being - Use regenerative farming to manage the landscape - Support farmers to produce more local products - Raise public awareness of any changes | 1. Mosaic of linked habitats- biodiversity 2. Reintroduction of species richness – eg red squirrels 3. Enhanced inclusive community engagement- wellbeing 4. Improved access for all- wellbeing 5. Local prosperity- economic benefits | 1. Re-wetting grassland areas and Rhos pasture- in buffer around streams/rivers 2. Reduction in sheep through financial incentives or compensation – on in bye land 3. More cattle farming on rhos pasture 4. Less bracken on upland slopes- more heather 5. Increase hay meadows – expand current hay meadows | 1. Peatland and low carbon farming 2. Other Trees 3. Woodland creation- natural regeneration 4. Ffridd 5. Scrub 6. Semi-natural grassland | - Peatland-maintain and restore in upland peat areas. - Low carbon farming on all farms - Corner of field margins and hedgerows for ‘other trees’ - Natural regeneration along rivers and streams and to extend current woodlands - Expand ffridd from current areas through natural regeneration |
| Land |  | - Water - Biodiversity - Carbon - Access - Community | - Sustainable economic continuous cover forestry (timber security) - Hydropower- link up to local network | 1. Peatland restoration 2. Woodland creation 3. Agroforestry/Wood pasture/other trees (in-bye) 4. Scrub/ffridd 5. Alter grazing species (more ponies and cattle) | - Peatland- re-wet hydrological function (deep and shallow peat) - Scrub at the bracken line where woodland should be - Scrub around edges to soften transitions - Woodland creation near to water courses (riparian zones) - Woodland creation near to existing (connectivity) - Keep conifer (not more) and shift to mixed broadleaf - Fringe areas around forestry blocks- soften edges and less hard lines - Allow small and scattered trees (willow) on peat as part of natural regen processes - No planting where curlew have been found (ring fence existing population) and not in the upland valley area towards the North west of the area |
| Conservation | - Bigger and better, connected woodland - Mosaic of habitats in good ecological condition - More hydro and solar energy - Nature friendly farming- winter grazing in woodlands and more mixed livestock, support regen - Sustainable incomes- farming, forestry, wider landscape - Good Bog - Farming landscape | - Biodiversity - Climate change - Water Quality - Cultural- peoples heritage - Economy and livelihoods | - Reduced winter grazing through subsidies to improve the quality of in-bye - Increase cattle numbers in winter- could need more woodland | 1. Peatland-rewetting 2. Keeping hedges and edges- increase- allow to flower, fruit, cutting rotation 3. Prioritise connectivity 4. Good seed sources- regen appropriate areas 5. Saber planting- Ffridd development in areas inaccessible by sheep and browsers- going up craggs. More FFridd in transition areas that are currently one use of grazing 6. Cattle woodland winter- dependent on others 7. Some increasing of meadows for carbon capture of flowers   rotation- mob grazing- rotation of sheep for 10years to bring back trees | - Curlew- 1km no tree area around known curlew locations, in-bye with hedgerows - No planting on active bogs- natural regen okay, as long as restoration parts are wet - No trees on SSSI flower meadows - No conifer planting- unless ecological the land is of low ecological score - Case by case use of in-bye land and development of treescapes - Do not plant across farms in back-to-back blocks - No vegetation on lead mines - No fencing of uplands |
| Farming |  | - Peatland – carbon and water management - Food- passion for local meat, nutritional value, biodiversity - Wildlife - Culture and Farming Heritage of the landscape | - Peatland restoration - Some more Scots Pine - Only planting what belongs here - People as part of the ecology - Grazing in wood pasture (in existing woodlands) - Fridd trees- natural regeneration - Scrubby habitats - Rhos pasture - Trees for shelter for livestock- shade and animal welfare - Small plots of trees - Avenues of trees around buildings – keep the heritage by succession planting replacements for old heritage trees. - More diversity - Good species rich grassland | 1. Peat restoration 2. Species rich grassland 3. Ffridd/Scrub   Rhos pasture  Shelter trees   1. Remove some conifers around the neighbouring landscape to reduce the wider seed source | - No tree planting on peat but allow any that naturally come up like willow - Create a buffer to not allow natural regen around watercourses to protect water voles - Keep sheep grazing overwinter - Allow natural regeneration on peat and ffridd - Scattered trees must be around buildings and tracks to replace heritage ones lost - No trees on rhos pasture or species rich grassland - Scattered trees only on the edges no in-field trees |
